# CRISPR-Cas9基因编辑技术在范可尼贫血中的研究进展

**DOI:** 10.3760/cma.j.cn121090-20240825-00321

**Published:** 2025-03

**Authors:** 怡曼 高, 丽贤 常, 晓凡 竺

**Affiliations:** 1 中国医学科学院血液病医院（中国医学科学院血液学研究所），血液与健康全国重点实验室，国家血液系统疾病临床医学研究中心，细胞生态海河实验室，天津 300020 State Key Laboratory of Experimental Hematology, National Clinical Research Center for Blood Diseases, Haihe Laboratory of Cell Ecosystem, Institute of Hematology & Blood Diseases Hospital, Chinese Academy of Medical Sciences & Peking Union Medical College, Tianjin 300020, China; 2 天津医学健康研究院，天津 301600 Tianjin Institutes of Health Science, Tianjin 301600, China

## Abstract

范可尼贫血（FA）是一种以基因组不稳定和对DNA交联剂敏感为特征的遗传性骨髓衰竭综合征。近年来，CRISPR-Cas9技术在FA的基因治疗领域取得了突破性进展。传统CRISPR-Cas9技术在FA基因编辑中已得到成功应用，单碱基编辑技术基于CRISPR/Cas9系统，能够对FA患者中常见的基因突变进行精准、高效的基因修复；尽管引导编辑技术提供了新的基因编辑可能性，但目前在FA中的应用尚未开展。FA基因编辑技术虽取得了显著进步，但仍面临诸多挑战，包括收集足够的造血干细胞、基因编辑后增加肿瘤发生风险、染色体不稳定、脱靶效应等。未来研究应聚焦在优化单链向导RNA和Cas9核酸酶、设计更严格的PAM序列方式等减少脱靶效应，设计个性化的基因编辑策略。伦理与监管问题和长期随访同样也是今后开展基因编辑工作的重点。随着技术的不断进步和临床试验的深入，我们有望在未来看到CRISPR-Cas9技术在FA治疗中取得更多的突破。本文就常用的CRISPR技术在FA治疗领域的最新研究进展展开综述，并分析了该技术在FA基因治疗中的优势与挑战。

## Abstract (en)

Fanconi anemia（FA）is a hereditary bone marrow failure syndrome that is characterized by genomic instability and heightened sensitivity to DNA cross-linking agents. In recent years, the CRISPR-Cas9 technology has exhibited groundbreaking progress in the field of gene therapy for FA. The traditional CRISPR-Cas9 technology has been successfully applied in FA gene editing. Further, single-base editing technology, based on the CRISPR/Cas9 system, performs precise and efficient gene repair for prevalent gene mutations in patients with FA. The prime editing technology provides new possibilities for gene editing; however, its application in FA has not been initiated. Despite significant advancements in FA gene editing technology, several challenges remain, including the collection of sufficient hematopoietic stem cells, the risk of increased tumorigenesis postgene editing, chromosomal instability, and off-target effects. Future research is recommended to focus on optimizing sgRNA and Cas9 nucleases, designing stricter PAM sequences to reduce off-target effects, and devising personalized gene editing strategies. Further, ethical and regulatory issues as well as long-term follow-ups are crucial priorities for future gene editing work. With continuous technological advancements and in-depth clinical trials, we expect more breakthroughs in FA treatment using the CRISPR-Cas9 technology in the future. This article reviews the latest research progress of CRISPR technology in FA treatment and analyzes the advantages and disadvantages of this technology in FA gene therapy.

范可尼贫血（Fanconi anemia, FA）是一种以常染色体隐性遗传、常染色体显性遗传（FANCR/RAD51）或X连锁（FANCB）遗传的骨髓衰竭疾病，具有先天性发育异常、进行性骨髓衰竭和肿瘤易感性等临床特征[1]。FA/BRCA通路主要负责识别DNA链间交联（interstrand cross-linking, ICL）并参与同源重组修复（homology directed repair, HDR）[2]–[3]，该通路23个基因中的任何一个发生突变都可能导致FA发病[4]–[5]。在所有已知的基因突变中，FANCA基因的双等位基因突变最为常见，约占所有FA患者的60％[6]–[7]。

造血干细胞移植（hematopoietic stem cell transplantation, HSCT）仍然是目前治疗FA患者唯一的根治性方法，3年生存率约为80％[8]–[9]。然而，因HLA匹配供者有限，故并非所有患者都有条件接受HSCT。此外，移植物抗宿主病（GVHD）[10]–[11]和移植后远期实体瘤[12]–[13]使部分患者无法从HSCT中获益。因此，针对FA患者的发病机制，最理想的治疗方案是通过基因治疗修复突变基因。与HSCT相比，基因治疗可为无合适HLA供者的患者提供新的方案，亦可最大程度减少移植后排异及远期实体瘤的发生，从而显著提升患者的生活质量。但由于FA患者造血干细胞（HSC）数量较少、基因编辑技术限制，导致FA基因治疗不能在临床广泛应用。2023年末，首个基于CRISPR的基因编辑疗法CASGEVY（exa-cel）[14]获批，标志着基因编辑技术正式进入了一个新时代。本文就CRISPR-Cas9技术在FA中的研究进展作一综述。

一、CRIPSR-Cas9基因编辑技术的作用机制

CRISPR-Cas9系统源于细菌和古细菌为应对外来入侵而进化出的防御机制[15]，主要通过单链向导RNA（single guide RNA, sgRNA）和Cas9蛋白组成。sgRNA包括CRISPR RNA（crRNA）和反式激活crRNA（tracrRNA），负责引导Cas9蛋白靶向特定的DNA序列。Cas9蛋白含有HNH和RuvC两个关键结构域：HNH切割与sgRNA互补的链，RuvC结构域切割非互补链。Cas9在sgRNA引导下，识别前间区序列邻近基序（protospacer adjacent motif, PAM），并在PAM位点上游3~5个碱基处切割DNA，产生双链断裂（double strand breaks, DSB）。双链断裂后细胞将进一步进行修复，修复机制主要包括非同源末端连接（NHEJ）和同源重组修复（HDR）两种方式。NHEJ是较常见的修复途径[16]，可以快速融合两个断裂的DNA末端（图1），但容易在靶位点发生随机插入或缺失，导致基因失活；HDR则利用同源供体DNA，能实现精准的插入、缺失或碱基置换，但效率较低[17]–[18]。HDR依赖功能性FA途径，FANCD1/BRCA2、FANCN/PALB2、FANCO/RAD51C、FANCR/RAD51、FANCS/BRCA1和FANCU/XRCC2等多个FA相关基因都发挥着重要作用[19]。

**图1:**
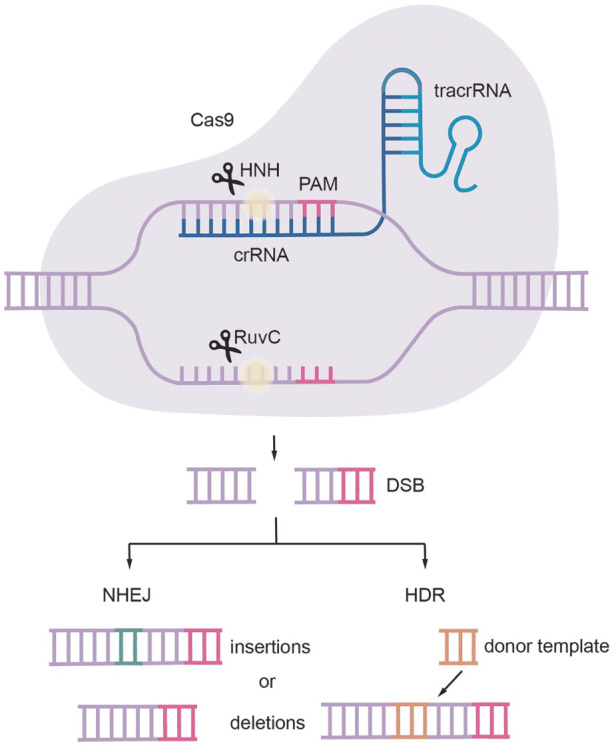
CRISPR-Cas9基因编辑技术原理 **注** Cas9：CRISPR相关蛋白9；PAM：前间区序列邻近基序；crRNA：CRISPR RNA；tracrRNA：反式激活crRNA；RuvC和HNH：Cas9蛋白中切割核酸的结构域；DSB：双链断裂；NHEJ：非同源末端连接；HDR：同源重组修复；insertions：插入；deletions：缺失；donor template：供体模板

二、CRISRR-Cas9技术在FA治疗中的应用

1. 传统的CRISPR-Cas9技术在FA中的应用：CRISPR-Cas9技术在FA基因治疗中取得了一些突破性进展（表1）。

**表1:** CRISPR-Cas9在范可尼贫血中的应用

年份	参考文献	靶基因	细胞来源	基因编辑技术	修复途径
2015	Osborn等[20]	FANCC	fibroblast	CRISPR-Cas9	HDR
2016	Osborn等[21]	FANCI	iPSC	CRISPR-nCas9	HDR
2017	Skvarova Kramarzova等[22]	FANCD1	fibroblast	CRISPR-Cas9	HDR
2019	van de Vrugt等[23]	FANCF	mESC	CRISPR-Cas9	NHEJ/HDR
2019	Román-Rodríguez等[24]	FANCA、FANCB、FANCC、FANCD1、FANCD2	CD34^+^细胞	CRISPR-Cas9	NEHJ
2022	Sipe等[25]	FANCA	HSPC	CBE、ABE	
2022	Siegner等[26]	FANCA	HSPC	ABE8e	

**注** CRISPR-nCas9：成簇规律间隔短回文重复/核酸酶缺失型Cas9；fibroblast：成纤维细胞；iPSC：诱导性多能干细胞；HDR：同源定向修复；mESC：小鼠胚胎干细胞；HSPC：造血干/祖细胞；NEHJ：非同源末端连接；CBE：胞嘧啶碱基编辑器；ABE：腺嘌呤碱基编辑器；ABE8e：腺嘌呤碱基编辑器8e

Osborn等[20]首次在FA患者成纤维细胞中，通过补充FA相关的DNA修复途径并结合嘌呤霉素抗性筛选，成功纠正了FANCC基因的c.456+4 A>T突变，基因编辑效率约2％，这是CRISPR-Cas9技术在FA基因编辑中的首次成功应用。在后续的研究中，他们又在FA患者的诱导性多能干细胞（induced pluripotent stem cells，iPSC）中，利用nCas9（D10A）和DNA质粒作为供体模板，对FANCI基因的c.1461 A>T突变进行了基因校正[21]，通过三轮丝裂霉素C（mitomycin C, MMC）筛选，基因编辑效率达到了66％，经基因校正后的iPSC成功分化为具有HSC特性的CD34^+^CD38^-^细胞。Skvarova Kramarzova等[22]研究团队则从1例患有复合杂合突变（886delGT和6162insT）的FA患者的皮肤活检样本中提取成纤维细胞，设计了单链寡核苷酸（ssODN）作为HDR模板，通过CRISPR-Cas9系统修复了FANCD1基因的886delGT突变，利用PARP抑制剂进行细胞筛选后，基因编辑效率约为23％。

尽管HDR介导的基因编辑在FA细胞中取得一定的成功，但其基因编辑效率普遍较低，且需要特定的筛选策略。因此，研究者开始探索NHEJ路径在FA细胞中恢复FANC蛋白功能的可能性。van de Vrugt等[23]利用CRISPR-Cas9基因编辑技术，对FA患者的FANCF基因突变进行了深入研究。他们发现，通过CRISPR-Cas9诱导的DSB触发NHEJ修复路径，无需外部模板，便可有效修复FANCF基因突变，经编辑的细胞在MMC的处理下存活率提升了27％。此外，该团队还在小鼠胚胎干细胞（mouse embryonic stem cells, mESC）中进行了HDR修复途径，尽管其基因编辑效率较低（≤6％），但经过基因编辑的胚胎干细胞在增殖方面展现出了明显的优势。在Román-Rodríguez等[24]的研究中，通过CRISPR-Cas9技术激活NHEJ机制，成功修复了FA患者HSC中的FANCA、FANCC、FANCD1等关键基因突变：利用CRISPR-Cas9系统在靶基因上引入DSB，利用NHEJ修复路径产生补偿性插入/缺失突变，恢复了基因的阅读框，从而纠正了突变基因的功能缺陷，经过编辑的细胞在体外（实验室培养）和体内（小鼠模型移植）实验中均展现出显著的增殖优势。

2. 单碱基编辑技术的应用：传统CRISPR-Cas9技术对单碱基突变的校正精准度较差，为了克服这方面的挑战，科研人员开发了单碱基编辑技术（base editing）。这项技术包括胞嘧啶碱基编辑器（cytosine base editor, CBE）和腺嘌呤碱基编辑器（adenine base editor, ABE）[27]–[29]，它们能够在不引起DNA双链断裂的情况下，利用与脱氨酶融合的nCas9，直接在目标基因位点实现C>T（G>A）或A>G（T>C）的碱基转换。相较于传统方法，这一技术更为精准、高效和安全。Moriarty实验室利用单碱基编辑技术成功修正了FANCA基因的突变，恢复了FANCA蛋白的正常表达，通过FANCD2的单泛素化验证了FA信号通路的完整，并且经过基因校正的细胞对MMC的抵抗力得到了显著增强[25]。Corn实验室则采用了改进型的ABE8e版本，优化了sgRNA的设计，成功修复了FANCA基因中的两种常见突变——FA-75和FA-55，实现了70％~80％的基因编辑效率[26]。

3. 引导基因编辑技术：引导基因编辑技术（prime editing）[30]是目前基因编辑领域的最新突破，通过其独特的“搜索与替换”机制，能够精确地执行所有12种可能的碱基编辑，包括置换、插入和缺失。这一技术超越了传统单碱基编辑器的局限，能够实现A到T或C到G的碱基颠换。这项技术尚未应用于FA治疗领域，但这种技术的发展为FA的治疗提供了新的方向。

三、CRISPR-Cas9技术的局限性及未来研究方向

CRISPR-Cas9技术在治疗FA方面展现出巨大的潜力，但其临床应用仍面临一些挑战。首先，收集足够的HSC是基因治疗成功的关键。研究表明，使用粒细胞集落刺激因子（G-CSF）和普乐沙福（plerixafor）动员HSC对尚未出现骨髓衰竭的FA患者是安全且有效的[31]，然而，大多数FA患者在确诊时已出现显著的全血细胞减少，使HSC的收集难度大幅增加[32]。提示我们对于FA患者应该在还未出现骨髓衰竭前或者在骨髓衰竭早期尽快收集HSC，这将有利于后期行基因治疗。其次，FA患者因DNA修复机制缺陷，CRISPR-Cas9引发的DSB难以有效修复，导致基因组大片段的缺失和染色体重排[33]–[34]，增加肿瘤发生风险，这种风险在具有长期自我更新能力的HSC中尤为显著。尽管单碱基编辑和引导编辑技术不产生DSB，然而编辑过程中产生的单链损伤仍可能引发染色体不稳定。此外，基因编辑技术的脱靶效应[35]也是影响其安全性的关键，特别是在DNA修复功能受损的FA患者中，脱靶效应可能诱发严重的基因组损伤。为提高基因编辑的安全性，未来研究应聚焦以下几方面：优化sgRNA和Cas9核酸酶、设计更严格的PAM序列、优化递送方式、限制核酸酶的表达时间及开发更精确的脱靶检测技术[36]等。今后是否可以通过寻找可通用的非FA患者靶细胞克服FA患者DNA修复机制缺陷值得我们探索。此外，针对FA患者不同的基因突变类型，基因编辑策略需个体化设计，以实现最佳治疗效果。对于常见点突变，单碱基编辑和引导编辑技术可实现高效的基因矫正；但对于复杂的突变或大片段缺失，传统CRISPR-Cas9可能更具优势。这就要求我们熟练掌握各种基因编辑技术的优劣，并根据突变情况合理选择。

临床治疗工作中，伦理与监管问题亦必须重视。在推进基因编辑技术的同时，须确保其应用符合伦理标准和监管要求。接受CRISPR-Cas9治疗的患者需进行长期随访，以评估潜在的长期风险[37]。这就要求我们设立专有机构进行监督与评估。

四、结语

CRISPR基因编辑技术作为革命性的基因编辑工具，在FA的治疗中展现出巨大潜力。尽管仍存在许多挑战，但相信随着基因编辑技术的不断优化，最终FA基因治疗将会在临床中得到推广。
